# Strategies for ensuring quality data from Indian investigational sites

**DOI:** 10.4103/2229-3485.80367

**Published:** 2011

**Authors:** Antal K. Hajos, Sujal K. Kamble

**Affiliations:** *Managing Director, Procelsis Consulting, Germany*; 1 *Associate Partner Asia-Pacific, Procelsis Consulting, Germany*

**Keywords:** Clinical monitoring, clinical trial, compliance, data quality, investigational site, PDCA, site management, site selection

## Abstract

The topic of ensuring quality and compliance is and must be a top priority in the conduct of clinical trials, as warranted by regulatory guidelines as well as the inherent responsibility of the professionals conducting such research. Fast-growing emerging clinical geographies such as India demand special attention due to rapid growth and associated factors that may put study quality at risk. In this paper, we used the basic principle of PDCA (Plan, Do, Check, and Adjust) to structure the processes of a clinical trial from protocol to final analysis in order to highlight the interactive nature of involved people and processes required to ensure quality of data and site functioning.

## QUALITY MANAGEMENT AND DATA QUALITY

Quality in clinical research may be defined by the reliability and credibility of information and data collected. Such information must provide an answer to a scientific question, and the trial process applied must be in compliance with defined (scientific and regulatory) requirements.[1]

The principles of Quality Management Systems have since decades been applied to the conduct of clinical trials and are woven into the regulatory guidelines, ICH GCP, and associated requirements. The conduct of a multinational and multicenter trial is a highly complex endeavor. Herein, we used the basic principle of the PDCA cycle (Plan, do, check, and adjust), initially introduced by Deming,[2] to structure the key steps at which quality-relevant interactions occur. Although the PDCA cycle has been utilized herein for sake of simplicity, DMAIC is the more modern and accepted standard.[3] The DMAIC project methodology has the following five phases:

- *Define* the problem, the voice of the customer, and the project goals, specifically.
- *Measure* key aspects of the current process and collect relevant data.
- *Analyze* the data to investigate and verify cause-and-effect relationships. Determine what the relationships are, and attempt to ensure that all factors have been considered. Seek out root cause of the defect under investigation.
- *Improve* or optimize the current process based upon data analysis using techniques such as design of experiments, poka yoke or mistake proofing, and standard work to create a new, future state process. Set up pilot runs to establish process capability.
- *Control* the future state process to ensure that any deviations from target are corrected before they result in defects. Implement control systems such as statistical process control, production boards, and visual workplaces, and continuously monitor the process.

**Figure 1:**
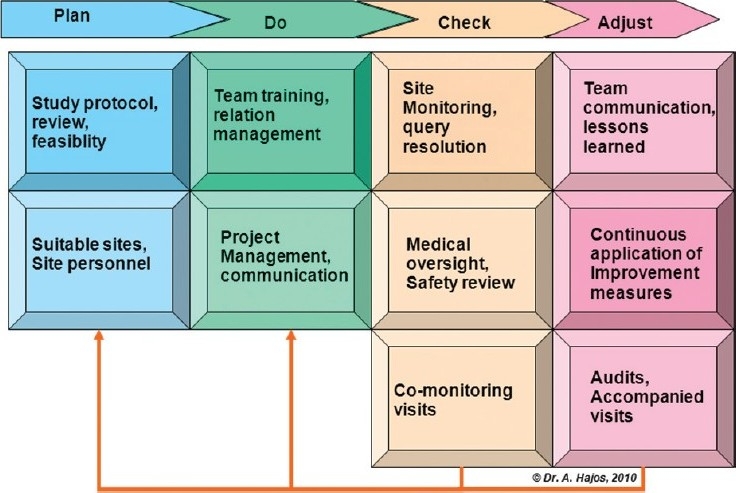
Key elements of trial conduct under a Plan, Do, Check, and Adjust structure

Both, PDCA and DMAIC ultimately highlight key elements of quality management: Ensure training, ongoing observation/checking/monitoring, continuous improvement, and independent audit. We believe that such framework provides for a beneficial system to analyze and manage requirements leading to superior quality of clinical trial conduct and data from investigational sites. Figure 1 shows a simplified overview of the key quality levers involved in a clinical trial, structured according to PDCA. These are discussed in more detail below.

Although India still carries a minor load of patient recruitment on a global scale, it is among the strongest growing markets for clinical trial conduct and has itself established on the forefront of emerging, attractive trial locations. The fast growth of the clinical trial industry, which is little older than a decade in India, brings about a certain lack of experienced personnel in clinical organizations (such as from pharmaceutical companies or contract research organizations (CROs)) as well as investigational sites.[4] High demand due to strong growth amplifies the shortages in experienced sites with sufficient infrastructure, as well as breadth of training.[4] Add cultural attitudes of hierarchies as well as a strong business pressure, and the result is a geography with extremely high potential, yet also the need for special oversight in terms of ensuring quality.[5] Below discussion is set to structure the key factors in managing and maintaining quality at sites and data at the key interfaces of people and processes involved.

## THE PLAN-PHASE: STUDY PROTOCOL AND FEASIBILITY

In the planning phase, quality management of data from sites actually starts at the sponsors’ site with the protocol design. Although a review of proper protocol design would be beyond the scope of this article, it is important to note that the feasibility, soundness, and consistency of a protocol is key to any of the subsequent steps ensuring quality. In particular, it is important that a protocol is written such that protocol procedures actually reflect medically and logistically current and sound practice of the geographies and sites to be participating in the study. Having said this, it also needs to address the aforementioned scientific and regulatory questions. Often, it is that very alignment of these two aspects proving most difficult in separating good from poor protocols. Knowledge of the indication and previous trial experience are probably the strongest factors ensuring high quality in this regard.

## The DO-PHASE: STUDY FEASIBILITY AND SITE SELECTION

A further practical verification, i.e., feasibility study, of the protocol is probably indicated in any case. We have previously reported on study feasibility and site selection in India[6] and readers would be referred to that article for a more detailed process description.

Every protocol is unique in itself; therefore, for every new study, the criteria and tool(s) applied for selection of site would vary. A site that is well suited for one study is not necessarily good for the other. Some sites may have been on favorite lists of organizations for any and every study. This may lead to mining suboptimal data from excellent sites. The right selection of the site is therefore one of the first steps of quality conduct. But, how then do we best rate sites? An investigator database, preferentially tracking previous performance and issues is a standard and highly valuable tool for initial selection and review of sites. Additionally, a systematic rating tool should be utilized for every study with every new and also known site. A simple excel sheet may be a good start to record the qualified sites, e.g., by rating them in terms of experience and qualification of Principal Investigator (PI) and his/her team, patient pool, etc. for the given protocol. Some factors for consideration of site qualification are shown in Table 1 but should be extended to reflect the specifics of the protocol under question and issues likely to arise. These could be medical practice, logistics, nature and availability of patients, recording needs and issues, special technology employed and its standardization.

Aside of the medical practice and patient pool, availability of staff resource for the study tends to be a frequent concern at sites. If supported by the study budget, appointing a Site Management Organization (SMO) is a worthy consideration, provided the SMO is highly efficient and operates in a thorough, professional approach. This approach may be particularly beneficial for complex studies or inpatient studies mandating high site resources and administrative support. A CRO or the sponsor itself may alternatively provide such targeted site support.

The experience and expertise of the project manager is a further important factor. As the key coordinator at the local and/or global level, the project manager is the prime source for identifying issues that may arise, and foster an environment of transparency and openness to initiate preventive and corrective actions. The project manager may not have to be the primary expert on all aspects of the trial, as he would be supported by specific functions such as Regulatory Affairs and Quality Assurance. The project manager is however key to communication among all stakeholders, providing the overarching communication layer to ensure detection and improvement measures.

Finally, appointment of a highly experienced and proactive coordinating investigator who may mentor and counsel other sites in the country or region may be a valuable measure to ensure consistency and support where it is needed. This would be important also in light of the regional differences in medical practice and patient profiles.

**Table 1:** Factors at sites likely to impact data quality (examples)

Factor class	Description
Compliance	Documention compliance, and practical application and adherence to procedures and practices at site.
Data accuracy	Patient database at site, as well as level and quality of data maintenance of it for at least the past 5 years.
Efficiency	Actual patient pool related to protocol requirements.
Motivation	Staff management by investigator
Motivation	Sincerity of the Principal Investigator and support staff involved.
Compliance	Training and ongoing guidance provided by the sponsor and/or CRO for current and previous protocols.
Compliance and training	Quality awareness and quality management system components (such as training, Standard Operating Procedures) implemented at the site.

## THE CHECK: MONITORING, DATA MANAGEMENT, MEDICAL OVERSIGHT, AND SAFETY REPORTING

Site monitoring is at the heart of study conduct and the primary, site-independent check of site- and data quality, and compliance. Note that monitoring is a key in-process quality control procedure, but not an auditing procedure. This also does not preclude that a site itself would and should have certain quality control measures implemented for their internal quality check.

Trial monitoring, as defined in ICH GCP, E6, 5.18,[7] is primarily to verify that:

- The rights and well being of human subjects are protected.
- The reported trial data are accurate, complete, and verifiable from source documents.
- The conduct of the trial is in compliance with the currently approved protocol/amendment(s), with GCP, and with the applicable regulatory requirement(s).

The reader is also referred to ICH E6, Section 5.18.4 on “Monitor’s Responsibilities,”[8] which provides a comprehensive listing of the specific action to be taken to verify and ensure appropriate trial conduct at the site.

In terms of preventive action as part of the monitor’s responsibility, she or he should focus on potential factors at the site that hamper or affect the quality standard. Ideally, such factors are detected, discussed, and eliminated before impacting the data (“preventive”) rather than afterwards (“corrective”). A well-trained and alert monitor can usually identify such factors during the first few patient cases, and initiate a lesson learned approach, as appropriate, in alignment with the project manager. It is also important to pool the initial experiences and learning during a study among all stakeholders. To this end, group discussions among the monitoring team are also an important aspect that helps Clinical Research Associates (CRA/monitor) to serve the site in a successful way. Discussing major issues that had occurred at individual sites globally by the project manager or e.g. Lead CRA would make other monitors alert in areas they otherwise may miss. Establishing a database or platform to track and communicate frequently asked questions that are accessible to the entire team has proven to be another useful tool.

Good monitoring makes an auditor’s job easier and, more importantly, creates a certain comfort and transparency for everybody what the outcome of an audit would likely be. As always, a CRA, like everybody else in the trial, should take an attitude of welcoming any suggestions and findings for improvement during an audit, rather than trying to hide or prevent findings. Well-conducted site audits are amongst the most valuable learning experiences a CRA may have. The same actually also applies to accompanied compliance visits which unfortunately seem still to be either not conducted at all or at low frequency in most sponsor and CRO organizations. Likewise, internal audits by the sponsor and/or CRO should be considered a standard tool in the quality conduct of clinical trials, rather than the exception.

If the study is of longer duration, it is important to arrange refresher trainings periodically for the site staff. Such meetings or training events are also keys to ensure ongoing motivation, a lack thereof again being expected to decrease attention to quality at the site.

Finally, high-quality monitoring should be done with the same care from the first, to the last line of last case report form (CRF). The rights and well being of the study subjects must always be paid full attention as foremost responsibility, as spelled out in the ICH GCP guidelines. Source data verification hence needs highest attention.

Data management and query resolution are other important aspects of ensuring quality data from and at sites. There has to be perfect coordination, cooperation, and short turnaround times between the monitoring- and the clinical data management (CDM) team. Lack of alignment and clarity at this often somewhat difficult, yet essential functional interface that can negatively affect the data quality. Both, the data management and clinical research teams should have a full understanding about individual responsibilities and operational knowledge of each other’s profiles. Standardization, solid automated procedures, communication standards and plans, and medical and project management oversights are the tools to optimize this interface.

For example, it is important to get timely data, that is, CRFs need to be collected in a timely fashion to have the data entered into the clinical database, and query issues quickly. Only then can they be corrected soon after occurrence, and learning from these can be used for further cases. Early queries from CDM also reduce the often tremendous load of data management and monitoring efforts in the later stage, rushing at the end.

Unnecessary and illogical queries lead to irritation, lost interest, and lack of cooperation from sites. Therefore, there should be a logical, defined, and standardized way to handle query resolution agreed upon upfront among the entire team (e.g., in the data management plan).

The monitoring manual, the data management plan, and documentation of lessons learned are key and standard tools that go a long way to ensuring alignment and quality consciousness. Additional aspects are a medical oversight responsibility woven into the data collection and analysis process, and medical review of case data, where applicable. Finally, a safety plan, process, and system need to be established to ensure proper pharmacovigilance reporting as per regulatory requirements. Having multifunctional responsibilities defined and aligned, quality data will be the result.

Below, two practical examples of quality issues at sites and their root cause analysis are provided.

### An example of a site failing to deliver sufficient quality for a respiratory protocol

At initial assessment, the site appeared to be well suited:

- Investigator was qualified, trained, and research oriented
- Staff was highly qualified, trained, and sincere
- Site was well equipped
- Data maintained were good.

Despite all these criteria fulfilled, of 9 patients enrolled, 7 were either a wrong enrollment as per protocol criteria, wrong randomization, or inappropriate drug dispensing method followed. The site was excluded from future studies due to the following reasons:

- Investigator’s interest was superficial. Investigator was adamant to accept the flaws
- No practical application of SOPs at site
- Research officer was highly qualified but confused about the protocol procedures
- Repeated major errors occurred, despite prompt alerts and warnings
- Overall casual approach toward the study.

In this example, the main reason for failure is likely to be the investigators’ insufficiently sincere approach to the study. Investigators practically will not be able to always pay 100% attention due to their busy schedule. It is yet required that investigators ensure the necessary attention and priority within the study team to provide quality output and strict adherence to protocol. This management ultimately is a key element of the PI’s responsibilities.

### Example of failed monitoring that may have led to below standard data quality

The site was experienced and fulfilled criteria of a good site.

The monitor appointed by a CRO was sincere and hard working with some basic experience. The site partly failed for the following reasons:

- Monitoring was extremely slow due to change of CRAs during the initial trial period.
- High staff turnover at CRO caused discomfort and weak rapport between monitors and investigator’s team.
- Monitor finally appointed was rude and overly confident to challenge highly experienced and qualified investigator.
- Due to unsubstantiated suspicion, repeated audits were conducted at that site, which caused increasingly lack of interest and slow recruitment from the site.

The best possible balance of quality management and relation management between the sponsor, CRO as applicable, and the Investigators site needs to be maintained and managed in order to ensure continuous commitment of all involved, and is a prerequisite for quality research conduct. Optimizing motivation, awareness, and quality consciousness is key to successful teams. Clearly, personal relations between monitors and site personnel, commitment by the sponsor for ongoing collaboration beyond one trial, and ultimately the development of trust and appreciation play a major role.

## SUMMARY

Honesty, sincerity, and transparency are key requirements of long-lasting success in any business endeavor. Clinical research is one of the most regulated and delicate assignments for a team to handle. To build trust with the site staff, the sponsor must follow a true team approach across all stakeholders. Ego should be kept at the bay and there have to be optimized interpersonal relationship between the sponsor’s team, the CRA staff (at the sponsor and/or CRO), the SMO if applicable, and the investigator’s team. Additionally, there need to be a dedication and continuous efforts to strive for best results.

We have discussed herein that for every study, the available tools and practices need to be tailored to the specific protocol needs. Indication-specific SOP- and protocol training is a key factor in ensuring that everybody is aware of their own responsibilities and the comprehensive framework of the study. Training should be extended to include co-monitoring training visits as well as accompanied compliance visits. Audits are used to verify the suitable process-framework and adherence to it, from a regulatory and Quality Management perspective.

A high alignment of investigational site personnel, monitoring staff, and all other responsible at the sponsor and CRO are a key success factor, where project management, in alignment with the training and QA function, ensure communication and continuous improvement. Using such principles, highest quality data can be produced in a clinical trial from an emerging region such as India, where the team jointly can compensate factors such as high work pressure, high patient load, partial lack of experience, and logistic and infrastructural challenges.
